# Evaluation of renal replacement therapy in children and adolescents in the state of Amazonas, Brazil

**DOI:** 10.1590/1984-0462/2022/40/2021057

**Published:** 2022-04-04

**Authors:** Ana Matilde Menezes Melik Schramm, Inalda Facincani, Fabio Carmona

**Affiliations:** a Universidade do Estado do Amazonas, Manaus, AM, Brazil.; b Universidade de São Paulo, Ribeirão Preto, SP, Brazil.

**Keywords:** Chronic kidney disease, Renal replacement therapy, Children, Amazon, Doença renal crônica, Terapia de substituição renal, Crianças, Amazônia

## Abstract

**Objective:**

To describe the characteristics of stage-5 chronic kidney disease (CKD) children and adolescents undergoing renal replacement therapy (RRT) in Amazonas, Brazil, estimating the frequencies of current and new cases, describing the presence of anemia and bone metabolism disorders.

**Methods:**

Thirty-five patients aged 7 to 19 years-old on hemodialysis (HD) or peritoneal dialysis (PD) were studied between June 2018 and April 2019. The frequencies of current and new cases were estimated based on the 0 to 19 years-old population of Amazonas, in the same period. Data were collected about the underlying cause and diagnosis of CKD, dialysis, and biochemical analysis.

**Results:**

The frequencies of current and new cases were 24 and 15 patients per million people of compatible age (pmpca), respectively. The causes of CKD were nephrotic syndrome (22.8%), nephritic syndrome (14.3%), and neurogenic bladder (14.3%); in 48.6%, the cause was unknown/not investigated. Ten patients underwent renal biopsy, seven with segmental and focal glomerulosclerosis. The majority (80%) were on HD, with an average kt/V of 1.4, and in 51.4% the vascular access was the double lumen catheter. Hypocalcemia was found in 82.8% of patients, hyperphosphatemia in 57.2%, vitamin D insufficiency or deficiency in 60%, and altered parathyroid hormone values in 48.6%. Hemoglobin was low in 80%, with absolute/functional iron deficiency in 28.6%.

**Conclusions:**

In children and adolescents of Amazonas, Brazil, we found 24 pmpca with stage-5 CKU currently in RRT and 16.3 pmpca per year of new cases requiring RRT. Most patients were adolescents on HD, half without a causal diagnosis of CKD, with a high frequency of anemia and bone metabolism disorder.

## Resumo

**Objetivo:**

Descrever as características de crianças e adolescentes com doença renal crônica (DRC) dialítica no estágio 5 submetidos à terapia renal substitutiva no Amazonas, Brasil, estimando a frequência de casos atuais e novos, e discorrer sobre a presença de anemia e distúrbios do metabolismo ósseo.

**Métodos:**

Foram estudados 35 pacientes de 7 a 19 anos em hemodiálise ou diálise peritoneal (DP) de junho de 2018 a abril de 2019. As frequências de casos atuais e novos foram estimadas com base na população do Amazonas de 0 a 19 anos no mesmo período. Foram coletados dados sobre a doença de base da DRC, seu diagnóstico, a diálise e a análise bioquímica.

**Resultados:**

As frequências de casos atuais e novos foram 24 e 15 pacientes por milhão de pessoas em idade compatível (pmpic), respectivamente. As causas da DRC foram síndrome nefrótica (22,8%), síndrome nefrítica (14,3%) e bexiga neurogênica (14,3%), com 48,6% de causa ignorada. Dez pacientes fizeram biópsia renal, sendo sete com glomeruloesclerose segmentar e focal. A maioria (80%) fazia hemodiálise, com Kt/V médio de 1,4, sendo o cateter venoso central de duplo lúmen o acesso vascular em 51,4% dos casos. Observou-se hipocalcemia em 82,8% dos pacientes, hiperfosfatemia em 57,2%, insuficiência ou deficiência de vitamina D em 60% e valores do paratormônio alterados em 48,6%. A hemoglobina encontrava-se baixa em 80%, com deficiência absoluta ou funcional de ferro em 28,6%.

**Conclusões:**

Em crianças e adolescentes do Amazonas, Brasil, encontramos uma frequência de 24 pacientes com DRC dialítica e 16.3 pmpic por ano de novos casos. Os pacientes são, principalmente, adolescentes em hemodiálise, metade sem diagnóstico causal da DRC, com alta frequência de anemia e distúrbio do metabolismo ósseo.

## INTRODUCTION

Chronic kidney disease (CKD) is an irreversible disease. Its diagnosis is based on the presence of structural or functional kidney damage or decreased kidney function, present for at least 3 months, regardless of the underlying cause.^1^ Glomerular filtration rate (GFR) is used for stratification of CKD in stages from 1 to 5, the latter being the most severe, in which renal replacement therapy (RRT) is needed. CKD is a public health problem worldwide, varying according to region, population, and context.^2^ In Brazil, in 2018, there were 133,464 patients in RRT, with prevalence and incidence of 23.5 and 6.0 patients per million population (pmp), respectively.^3^ In children, the incidence of CKD is about 50 times lower than in adults. However, at this period of life, the disease can lead to serious impairments in physical, psychological, and social development.^4^

According to the literature, congenital anomalies of the kidney and the urinary tract (CAKUT) are the main causes of CKD in children, with glomerulonephritis being the most common cause among adolescent.^5^ Kidney transplantation is the gold standard for RRT, and peritoneal dialysis (PD) is the preferred dialysis method over hemodialysis (HD) in pediatric patients. However, there is a great variation between RRT methods when developed and developing countries are compared.^5^ Bone metabolic changes related to secondary hyperparathyroidism (2HPT), anemia, and cardiovascular and psychosocial changes negatively impact the quality of life of pediatric patients.

In the state of Amazonas, Brazil, the large distances between the capital and the interior are a significant barrier for the early or timely diagnosis and adequate conservative management of CKD. The difficulty in performing renal biopsies and the small number of corrective urinary tract surgeries available in the Unified Health System (in Portuguese, *Sistema Único de Saúde*, SUS) compromise the etiological diagnosis. In the capital, the fragmentation of services that occurred in previous years made it impossible to accurately collect retrospective data on this disease; in the interior of the state, there are no data about CKD. Reliable information about these patients is essential for establishing public health policies for this population.

Thus, this study aimed to describe the characteristics of stage-5 CKD children and adolescents undergoing RRT in Amazonas, to estimate the frequencies of current and new cases, and to describe the presence of anemia and bone metabolism disorders.

## METHOD

This was a case-series study, carried out from June 2018 to April 2019. All patients undergoing RRT in three private outpatient clinics and one public hospital in Manaus (Amazonas, Brazil) were eligible for this study. The patients were selected from the RRT schedule system of each unit. All patients aged up to 19 years who were in a regular HD program, at least three times a week, or in a regular PD program, either ambulatory (CAPD), automated (APD), or intermittent (IPD), for at least three months, were included. Participants with irregular HD schemes in emergency rooms, those not able to communicate in Portuguese (mostly Indigenous), and those not consenting were excluded.

The study was approved by the Research Ethics Committee of the State University of Amazonas (UEA), protocol #2.474.997 and CAAE #80754517.4.0000.5016. Eighteen years-old and up patients, parents or legal guardians signed the Informed Consent Form containing all the necessary information about the research, and patients between 10 and 17 years-old also signed Informed Assent Forms.

The demographic data collected were: age, gender, self-reported ethnicity and place of origin, which kind of health professional made the diagnosis of CKD, and where it was made.

To estimate the frequencies of current and new cases of stage-5 CKD in children and adolescents in RRT, it was used as reference (denominator) the population of the Amazonas state between 0 and 19 years of age according to the Brazilian Institute of Geography and Statistics (in Portuguese, *Instituto Brasileiro de Geografia e Estatística*, IBGE, https://www.ibge.gov.br), which was of 1,590,622 inhabitants in 2018. The frequency of current cases was calculated as the number of patients aged 0 to 19 years undergoing RRT in Amazonas in 2018, divided by the number of people aged 0 to 19 years in Amazonas in 2018 (in millions). The frequency of new cases was calculated as the number of new cases of stage-5 CKD requiring RRT between 0 and 19 years-old presenting in the 11 months of the study, divided by the number of people between 0 and 19 years old from Amazonas in 2018 (in millions). The frequency of new cases was then annualized (i.e., divided by 11 and multiplied by 12). This estimate was possible because, in Amazonas, RRT is only offered in the city of Manaus.

Stage-5 CKD was confirmed by calculating GFR using the Schwartz formula,^6^ with results below 15 mL/min/1.73 m^2^, associated with ultrasonography (USG) of kidneys and urinary tract with signs of CKD and/or persistently elevated urea and creatinine concentrations.^7^ The underlying cause of CKD was identified through results of renal biopsies, laboratory tests of urine and blood, imaging tests, as well as reviewing medical records, when available.

Regarding RRT, the data observed were: type of RRT, time on RRT, type of vascular access in HD patients, adequacy of dialysis by calculating Kt/V, interdialytic weight gain, and blood pressure. For HD patients, the Kt/V was calculated using the online calculator for single-pool Kt/V in the Brazilian Society of Nephrology website (https://www.sbn.org.br/profissional/utilidades/calculadoras-nefrologicas/), with Daugirdas’ formula. In PD patients, it was not possible to calculate Kt/V due to local difficulties in collecting all the dialysis solution drained in 24 hours. According to the Kidney Disease Outcomes Quality Initiative (KDOQI) guidelines, the minimum Kt/V recommended for patients undergoing three HD sessions per week should be 1.2.

Anthropometric measures were collected using standard procedures. For HD patients, pre- and post-dialysis weight was measured for one week, and the average interdialytic weight gain value was used. PD patients were weighed with an empty abdominal cavity during the monthly consultation. The z-scores of weight and height for age and sex were calculated using the Anthro Plus software in the OMS website (https://www.who.int/toolkits/growth-reference-data-for-5to19-years/application-tools). Blood pressure was measured with a calibrated aneroid sphygmomanometer and standard techniques.

Except for pre- and post-dialysis urea, blood tests were collected with 12-hour fasting, in a non-dialysis day, in the same laboratory. Blood counts and serum iron, ferritin, transferrin saturation, total calcium, phosphorus, 25-OH-vitamin D, alkaline phosphatase, and pre- and post-dialysis urea were measured. The definition of anemia was based on the blood hemoglobin values recommended by the World Health Organization (WHO, 2011) according to each age.^8^ Values accepted as desirable for ferritin and transferrin saturation followed the 2012 KDIGO recommendations.^9^ For the reference values of calcium, phosphorus, vitamin D, and parathyroid hormone (PTH), the 2017 KDIGO references were considered.^10^ For alkaline phosphatase, reference values found in the CALIPER project (Canadian Laboratory Initiative in Pediatric Reference Intervals, https://caliper.research.sickkids.ca/#/) were accepted in children and adolescents, whose reference intervals were transferred to the Brazilian population by Fontes et al. in 2018.^11^

A formal sample size calculation was not performed since the plan was to study all subjects in the population of interest. As it was a case-series, observational study, descriptive analysis was used to characterize the studied population. The data was stored in Microsoft Excel spreadsheets (Microsoft Corporation, Redmond, USA), and central tendency measures (means, medians) and variation (standard deviations, quartiles, and interquartile intervals), or frequencies and proportions were used. Missing data were not imputed.

## RESULTS

Between June 2018 and April 2019, there were 39 patients under 19 years of age on RRT in Amazonas. Three 18-year-old patients were not included because they were irregularly submitted to hemodialysis in emergency rooms, according to urgent clinical and laboratory parameters; this happens when there are no vacancies for RRT in a specialized center, and, unfortunately, it is common. One patient was excluded because it was not possible to explain the ICF to his guardians due to the language barrier, as the family was indigenous and did not speak Portuguese. Therefore, 35 patients (89.7% of all eligible patients) in regular RRT were evaluated. The study started in 2018 with only 16 patients. However, along the 11 months of data collecting, we included 19 more patients, who started RRT and exceeded the 3-month threshold, in addition to the four patients already mentioned who were not studied. Thus, for estimating frequencies of current and new cases, all 39 patients were considered, although only 35 were studied. It was found a frequency of 24 patients per million population with compatible age (pmpca) currently in RRT and a frequency of new cases of 15 pmpca patients in the study’s duration (16.3 pmpca per year). The overall mean age was 14.3±3.0 years. The mean height-for-age z-score was -1.79±1.4 (minimum and maximum values of -4.99 and 0.91, respectively), being -1.9 the mean for girls, and -1.7 for boys. Table 1 depicts the population’s characteristics.

**Table 1.** Population’s characteristics.

	n	%
Gender		
Feminine	18	51.4
Self-declared ethnicity		
White	13	37.1
Non-white	22	62.8
Origin		
Capital	14	40.0
Interior	21	60.0
Place of diagnosis		
Emergency room	21	60.0
Ambulatory	8	22.8
Hospital	6	17.1
Who made the diagnosis		
Pediatrician	16	45.7
Nephrologist	11	31.4
Generalist	8X8	23.0

It was possible to identify the cause of CKD in 18 patients (51.4%) and, among the 17 patients (48.6%) with unknown etiology, old laboratory data or clinical history of 11 of them showed unquantified proteinuria (n=6), history of previous acute renal injury (n=3) or recurrent urinary tract infection (n=2); however, six patients had no complaints before the detection of CKD, despite the USG showing diminished and/or hyperechogenic kidneys. Only 10 patients underwent percutaneous renal biopsy. Table 2 shows the diagnostic data.

**Table 2.** Causes of chronic kidney disease.

	n	%
Cause of CKD	35	100.0
No diagnosis	17	48.6
Nephrotic syndrome	8	23.0
Nephritic syndrome	5	14.2
Neurogenic bladder	5	14.2
Renal biopsy	10	28.4
FSGS	7	20.0
MCD+IN	1	2.8
LN class IV	1	2.8
No glomerular tissue	1	2.8

CKD: chronic kidney disease; FSGS: focal segmental glomerulosclerosis; MCD: minimal change disease; IN: interstitial nephritis; LN: lupus nephritis.

Table 3 shows the characteristics of the dialytic treatment, in which hemodialysis and access by double-lumen catheter were predominant.

**Table 3.** Dialysis characteristics.

	n	%	Mean±SD	Minimum value	Maximum value
Time between diagnosis and dialysis			38.0±34.0	4.0	130.0
≥12 months	29	83			
Time in dialysis			13.0±15.0	3.0	66.0
≥12 months	25	71			
Dialysis modality					
Hemodialysis	28	80			
APD	5	14			
IPD	2	6			
Dialysis access					
Arteriovenous fistula	10	29			
DLC	18	51			
Tenckhoff catheter	7	20			
Kt/V			1.4±0.50		
Arteriovenous fistula			1.4±0.40	0.9	2.1
DLC			1.3±0.50	0.5	2.6
Weight gain (between sessions)					
≥1kg	10	29			
Hypertension	26	74			

SD: standard deviation; CKD: chronic kidney disease; APD: automated peritoneal dialysis; IPD: intermittent peritoneal dialysis, DLC: double lumen catheter; kg: kilograms.

The metabolic bone profile evaluation was partially compromised by the difficulty of collecting some tests. While all patients had a calcium measurement (corrected by serum albumin), phosphorus, and PTH intact, only 30 patients had their 25-OH-vitamin D level measured. Because of hyperphosphatemia, only 15 patients (43%) were using calcitriol.

Anemia was observed in 80% of the patients. However, ferritin and transferrin saturation values were abnormal in only 26 and 28% of patients, respectively. All patients were using erythropoietin. Absolute iron deficiency was identified in six patients (17%) and functional deficiency in four (11%). Metabolic bone and hematological parameters are shown in Table 4.

**Table 4.** Metabolic bone and hematological profile.

	Mean±SD	Minimum value	Maximum value	Unit
Metabolic bone profile				
Calcium	8.1±1.0	6.1	9.6	mg/dL
Phosphorus	5.8±2.2	2.3	12.9	mg/dL
25-OH-vit.D	26.0±10.0	4.5	46.7	ng/mL
Alkaline phosphatase	255.3±154.3	39.0	648.0	U/L
PTH	398.2±287.0	101.0	1065.0	pg/mL
Hematological profile				
Hemoglobin	9.6±2.7	6.0	17.0	g/dL
Hematocrit	29.6±7.9	19.5	51.8	%
Ferritin	297.4±260.6	8.7	1063.0	ng/mL
Transferrin saturation	28.5±15.4	6.0	67.0	%

SD: standard deviation; vit: vitamin; OH: hydroxy; mg: milligrams; dL: deciliters; ng: nanograms; mL: milliliters; U: units; L: liters; PTH: parathyroid hormone.

## DISCUSSION

In our study, the frequency of stage-5 CKD in the pediatric population undergoing RRT in the state of Amazonas was of 24 pmpca, while the frequency of new cases was 16.3 pmpca per year. High frequencies of anemia and bone metabolic disorders were also found.

Amazonas is the largest state in Brazil in territorial extension. Its flat topography, intersected by rivers, with few highways, makes river transportation the main means of commute for people, and those residing in the interior of the state (47%) can spend several days to reach the capital, Manaus, to get tertiary healthcare. The socioeconomic disparity between the interior and the capital leads to a concentration of all RRT services and all the 36 nephrologists (six of which are pediatric nephrologists) of Amazonas state in Manaus. Thus, it was possible to identify all 39 patients on RRT up to 19 years old in Amazonas.

In Brazil, Konstantyner et al. found, in 2012, a prevalence and incidence of pediatric patients in RRT of 20 and 6.6 pmpca, respectively, but with important differences between the Brazilian regions. The prevalence of stage-5 CKD in the North-Central West set was of 13.8 pmpca (the lowest in Brazil), while the incidence was of 5.5 pmpca.^12^ Likewise, incidence and prevalence are not uniform around the world. The prevalence in the USA (99.5 cases pmpca) contrasts with that of African countries (3.5 cases pmpca), reflecting the socioeconomic inequality. As more developed places provide access to earlier diagnoses and offer greater access to preventive and therapeutic measures, in others, the diagnosis is late, there is difficulty in registering and collecting data, and the access to conservative and dialytic treatment is restricted.^5,13,14,15,16^ This is the first study addressing this issue in the state of Amazonas alone. The frequencies of cases found here may reflect an improvement in care and diagnosis of CKD in pediatric patients over the past few years, although it is still below desired levels.

Most patients were adolescents and had been on RRT for about a year, but a few had been on dialysis for more than 12 months, and there was no reliable information about mortality or the number of patients who received kidney transplants before our study started. Konstantyner et al.^12^ also found a majority of adolescents (68.6%) in the pediatric population in RRT in the North-Central West set of Brazil.

The NAPRTCS study (USA) found a mean z-score for height of -1.49 in CKD children in 2011.^17^ In 2014, Harambat et al. found a mean z-score for height of -1.65 in patients from 20 European countries who started RRT before age 19.^18^ Our mean height-for-age z-score was expected for this population since the frequency of height deficits in children and adolescents is about three times higher in Brazil’s North than in South, Southeast, and Midwest.^19^

Worldwide, CAKUTs are the most frequent cause of CKD in childhood,^5^ but this study found a low frequency of CAKUTs. In almost half of the cases, it was not possible to establish a cause for CKD. In Manaus, there are two polyclinics and one outpatient clinic care for pediatric patients with kidney diseases in the SUS, in which pediatric nephrologists follow all kidney diseases, including CKD. Due to the lack of a pathologist with expertise in kidney diseases in Amazonas, renal biopsies materials are sent for reading in another state. Corrective surgeries by pediatric urologists in the SUS are scarce: there is a delay in the diagnosis since many patients from the interior seek care only in advanced stages of CKD, and, finally, some experience an adverse outcome before arriving in Manaus to undergo RRT. These data confirm previous Brazilian studies, which show disparities between regions, with the frequency of patients with CKD without a known cause in the Southeast (21.1%) much lower than in the North-Center West set (45.8%).^12^ The mentioned unfavorable conditions of transportation to medical facilities and socioeconomic disparity make the Amazonas region at major risk for CKD secondary to traditional and non-traditional etiologies, as well as chronic kidney diseases of unknown etiology (CKDu).^20^ This study shows the impact of inadequate care for children in the first years of life, since the low frequency of CAKUT may reflect the lack of early diagnosis.

Concerning RRT, the most common method was HD, and among those who underwent PD, two patients underwent IPD five times a week in the hospital for 12 hours, a modality that has been in disuse for several years in CKD. However, there is no chronic PD treatment funded by SUS^21^ due to factors that include high taxation, prohibitive freight costs, and far distances from large centers. Without vascular access for HD, nor SUS authorization for CAPD or DPA, chronic IPD was the only option to keep these patients on RRT. In Brazil, HD is much more frequent than PD in the pediatric population,^3,12^ although PD is the recommended dialysis method for children.^22^ On the other hand, in developed countries, kidney transplantation exceeds HD and PD, as in Japan (42% transplanted) and the United States (71% transplanted).^23^ Literature shows that children from the North and Midwest regions of Brazil have a four times lower probability of getting a deceased donor transplant than children from other regions.^24^ In Amazonas, the kidney transplant program, which included only adults, was suspended more than four years ago. Nowadays, patients who need kidney transplantation are referred to other states, like São Paulo and Ceará.

Among HD patients, the higher frequency of short- or long-term double-lumen catheter (DLC) compared to arteriovenous fistula (AVF) is justified by the difficulty in making the AVF in the SUS, in which the demand greatly exceeds the installed capacity, in addition to the small caliber of children’s vessels for making AVF. Although the average Kt/V is adequate, the value found in patients with DLC (1.37) was lower than in patients with AVF (1.46). In the literature, similar data shows that the average Kt/V of patients with DLC is usually lower than that of patients with AVF since the recirculation present in the DLCs can be circumvented in the AVFs.^25,26^

The obtained results for the bone metabolic profile showed hypocalcemia in more than three quarters of the patients and hyperphosphatemia in more than half of them, indicating poor control of the bone mineral disease. Less than half patients used calcitriol, due to high hyperphosphatemia frequency, and more than half of the patients had vitamin D deficiency or insufficiency, as well as PTH values below or above that recommended by KDIGO^9^ The lack of adherence to drug treatment and diet may have contributed to these results; however, fluctuations in medicines’ availability is common in Amazonas. In India, Desoky et al. found 83% of pediatric patients on RRT with high PTH, hypocalcemia, and hyperphosphatemia.^27^ In the 2018 Brazilian Dialysis Census, which also included the adult population on dialysis, 36% had inadequate PTH and 32% had hyperphosphatemia.^28^ Difficulty in controlling PTH levels is common in developing countries, where access to dietary phosphate binders is not universal. Adequate 2HPT control requires long-term follow-up, especially in growing patients. This study could not show fluctuations in bone metabolism parameters for these patients, but it did show the need for more careful monitoring.

Anemia was found in most patients, although all were receiving erythropoietin. Only in 28% of cases could anemia be attributed to iron deficiency. Factors such as malnutrition, folate deficiency, inadequate dialysis, and the presence of inflammation need further investigation. In Brazil, in 2018, 29% of all patients on RRT had anemia,^28^ and, even among Europeans and North Americans, the values vary between 50 and 80%.^29,30^

The strength of this study is to be the first in Amazonas to study the pediatric population in RRT, with a representative number of patients, although small, limiting its external validity. Among the limitations are the impossibility of obtaining the Kt/V of patients on PD and the absence of tests to further investigate anemia, associated with economic and access limitations. Besides, our results might have also been underestimated due to the fact that many Amazonian families are compelled to move to other states in search of treatment for their children’s kidney diseases.

This unfavorable situation for the pediatric population with CKD in the state of Amazonas could be mitigated with some measures, such as: training of general practitioners who work in the interior of the state; use of telemedicine, which could be an important tool for improving diagnosis and initial treatment, not only by general practitioners in the interior but also with an important role in history talking, physical examination, and referral to the capital, when necessary, by nurses and community health workers in remote locations where there are no physicians; creation of a network for the quick shipment of blood and urine samples to a location with an available clinical analysis laboratory for basic exams. Public authorities urgently need to take decisive measures to help this under-assisted population.

In conclusion, the frequency of pediatric patients with stage-5 CKD in RRT was 24 pmpca, and the frequency of new cases was 16.3 pmpca per year. Most patients are adolescents who have been on hemodialysis for an average of 13 months. There is no causal diagnosis of CKD in half of the patients, and the frequency of CAKUT is low, likely related to difficulties in establishing an early diagnosis. There is a high frequency of anemia, hypocalcemia, hyperphosphatemia, and changes in PTH levels, showing the need for urgent intervention by public health managers so that these patients receive better quality health care.

## References

[1] National Kidney Foundation (2002). K/DOQI clinical practice guidelines for chronic kidney disease: evaluation, classification, and stratification. Am J Kidney Dis..

[2] Glassock RJ, Winearls C (2008). The global burden of chronic kidney disease: how valid are the estimates?. Nephron Clin Pract..

[3] Neves P, Sesso RC, Thomé FS, Lugon JR, Nasicmento MM (2020). Brazilian Dialysis Census: analysis of data from the 2009-2018 decade. J Bras Nefrol..

[4] Romao JE (2004). Chronic kidney disease: definition, epidemiology and classification. Braz J Nephrol..

[5] Harambat J, Stralen KJ, Kim JJ, Tizard EJ (2012). Epidemiology of chronic kidney disease in children. Pediatr Nephrol..

[6] Schwartz GJ, Brion LP, Spitzer A (1987). The use of plasma creatinine concentration for estimating glomerular filtration rate in infants, children, and adolescents. Pediatr Clin North Am..

[7] Andrassy KM (2013). Comments on ‘KDIGO 2012 Clinical Practice Guideline for the Evaluation and Management of Chronic Kidney Disease’. Kidney Int..

[8] World Health Organization (2011). Vitamin and Mineral Nutrition Information System.

[9] McMurray JJ, Parfrey PS, Co-Chairs WG (2012). KDIGO Clinical Practice Guidelinefor Anemia in Chronic Kidney Disease. Kidney Int Suppl..

[10] KDIGO 2017 (2017). Clinical Practice Guideline Update for the Diagnosis, Evaluation, Prevention, and Treatment of Chronic Kidney Disease-Mineral and Bone Disorder (CKD-MBD). Kidney Int Suppl..

[11] Slhessarenko N, Andriolo A (2018). Transference of CALIPER total alkaline phosphatase reference interval to a Brazilian pediatric population. J Bras Patol Med Lab..

[12] Konstantyner T, Sesso R, Camargo MF, Santis Feltran L, Koch-Nogueira PC (2015). Pediatric chronic dialysis in Brazil: epidemiology and regional inequalities. PLoS One..

[13] Saran R, Robinson B, Abbott KC, Agodoa LY, Bhave N, Bragg-Gresham J (2018). US Renal Data System 2017 Annual Data Report: epidemiology of kidney disease in the United States. Am J Kidney Dis..

[14] Becherucci F, Roperto RM, Materassi M, Romagnani P (2016). Chronic kidney disease in children. Clin Kidney J..

[15] Orta-Sibu N, Lopez M, Moriyon JC, Chavez JB (2002). Renal diseases in children in Venezuela, South America. Pediatr Nephrol..

[16] Ladapo TA, Esezobor CI, Lesi FE (2014). Pediatric kidney diseases in an African country: prevalence, spectrum and outcome. Saudi J Kidney Dis Transpl..

[17] North American Pediatric Renal Trials and Collaborative Studies [homepage on the Internet] (2011). 2011 Annual Dialysis Report.

[18] Harambat J, Bonthuis M, Stralen KJ, Ariceta G, Battelino N, Bjerre A (2014). Adult height in patients with advanced CKD requiring renal replacement therapy during childhood. Clin J Am Soc Nephrol..

[19] Brazil - Ministério do Planejamento, Orçamento e Gestão [homepage on the Internet] (2010). Instituto Brasileiro de Geografia e Estatística - IBGE. Anthropometry and nutritional status of children, adolescents and adults in Brazil. Rio de Janeiro, 2010.

[20] Lv JC, Zhang LX (2019). Prevalence and disease burden of chronic kidney disease. Adv Exp Med Biol..

[21] amazonasnoticias.com.br [homepage on the Internet] (2019). Lyra K. Centro de hemodiálise pede socorro no Amazonas e pacientes podem perder tratamento.

[22] Verrina E, Cappelli V, Perfumo F (2009). Selection of modalities, prescription, and technical issues in children on peritoneal dialysis. Pediatr Nephrol..

[23] Nogueira PC, Feltran LS, Camargo MF, Leão ER, Benninghoven JR, Gonçalves NZ (2011). Estimated prevalence of childhood end-stage renal disease in the state of São Paulo. Rev Assoc Med Bras..

[24] Nogueira PC, Carvalho MF, Feltran L, Konstantyner T, Sesso R (2016). Inequality in pediatric kidney transplantation in Brazil. Pediatr Nephrol..

[25] Neves MA, Petnys A, Melo RC, Rabboni E (2013). Vascular access for hemodialisys: what’s new?. J Vas Bras..

[26] Borges PR, Bedendo J (2015). Risk factors associated with temporary catheter-related infection in patients on dialysis treatment. Texto contexto - Enferm..

[27] Desoky S, Farag YM, Safdar E, Shalaby MA, Singh AK, Kari JA (2016). Prevalence of hyperparathyroidism, mineral and bone disorders in children with advanced chronic kidney disease. Indian J Pediatr..

[28] Sociedade Brasileira de Nefrologia [homepage on the Internet] (2019). Brazilian dialysis census 2019.

[29] Behnisch R, Kirchner M, Anarat A, Bacchetta J, Shroff R, Bilginer Y (2019). Determinants of Statural Growth in European Children With Chronic Kidney Disease: findings from the cardiovascular comorbidity in children with chronic Kidney Disease (4C) Study. Front Pediatr..

[30] Chavers BM, Roberts TL, Herzog CA, Collins AJ, St.Peter WL (2004). Prevalence of anemia in erythropoietin-treated pediatric as compared to adult chronic dialysis patients. Kidney Int..

